# Enchanced levels of apolipoprotein M during HBV infection feedback suppresses HBV replication

**DOI:** 10.1186/1476-511X-10-154

**Published:** 2011-08-29

**Authors:** Jin-Gang Gu, Cheng-liang Zhu, Duo-zhi Cheng, Yan Xie, Fang Liu, Xin Zhou

**Affiliations:** 1 Center for Gene Diagnosis, Zhongnan Hospital, Wuhan University, Wuhan, 430071, P.R. China; 2 Department of Clinical Laboratory, Renmin Hospital of Wuhan University, Wuhan, Wuhan 430060, P.R. China; 3 Department of Clinical Laboratory of Taihe Hospital Affiliated to Hubei Medical College, Shiyan, 442000, P.R. China; 4 The State Key Laboratory of Virology, College of Life Sciences, Wuhan University, Wuhan 430072, P.R. China

**Keywords:** Hepatitis B virus, Apolipoprotein M, ELISA, RT-PCR, Replication

## Abstract

**Background:**

Chronic liver diseases can interfere with hepatic metabolism of lipoproteins, apolipoproteins. Hepatitis B virus (HBV) is a major etiological agent causing acute and chronic liver diseases. Apolipoprotein M (ApoM) is a high-density lipoprotein (HDL) apolipoprotein and exclusively expressed in the liver parenchyma cells and in the tubular cells of the kidney. This study was to determine the correlation between HBV infection and ApoM expression.

**Materials and methods:**

Serum ApoM levels in patients with HBV infection and in healthy individuals were measured by ELISA, ApoM mRNA expression were determined by RT-PCR, and the expression of S and E proteins of HBV, as well as the synthesis of viral DNA were measured by ELISA and real-time PCR.

**Results:**

The levels of serum ApoM was significantly elevated in patients as compared to healthy individuals (*P *< 0.001), ApoM promoter activity, mRNA and protein expression were all stimulated in cells transfected with infectious HBV clone. In addition, ApoM decreases the expression of S and E proteins of HBV and the synthesis of viral DNA.

**Conclusion:**

Raised ApoM levels in HBV infection may in turn suppress HBV replication, one of the protective mechanisms of nature.

## Introduction

Chronic hepatitis B virus infections are a worldwide public health burden. It is estimated over 400 million people to date are persistently infected and these chronic infections may progress to liver damage with cirrhosis and hepatocellular carcinoma [1-4]. HBV infection is generally considered to be noncytopathic, as chronic viremia is not accompanied by signs of hepatocyte damage. Instead, considerable evidence points to an important immune and inflammatory contribution to liver dysfunction[5-8].

Apolipoprotein M (apoM) is a recently discovered 25 kDa protein, which is mainly associated with high density lipoproteins (HDL) and a minor part is present in triglyceride-rich lipoprotein (TGRLP) and low density lipoprotein (LDL), it is structurally related to the lipocalin family[9-11]. However, the precise physiological functions of apoM are poorly understood at present.

As the liver is the key organ for the metabolism of lipids, lipoproteins and apolipoproteins, it is possible that the replication of HBV or expression of viral gene products affects hepatocellular function at the molecular level. For example, HBx can activate a number of cellular genes[12-14]. It may be hypothesized that the capacity of hepatocytes for apoM production could be changed when HBV infection occurs.

In our present work we have compared the expression of apoM among the serum of patients infected with HBV and healthy individuals. We have also tried to find out the mechanism that HBV effects ApoM production in cells and the roles of ApoM in the expression of HBs and HBe proteins of HBV and in the production of core-associated DNA of HBV were determined. We found that ApoM levels rise during the viral infection and that over expression of ApoM suppresses HBV replication.

## Materials and methods

### Subjects

One hundred and 126 chronic HBV-infected patients were enrolled in the study. The diagnosis of chronic hepatitis B was confirmed by the serological examination of HBsAg and the persistence of abnormal alanine aminotransferase activity for more than six months. We excluded patients who had any other types of liver damage such as autoimmune hepatitis, alcoholic liver disease, and Wilson's disease. The healthy control population consisted of 118 healthy unrelated individuals who were negative for HBsAg, anti-HBs and anti-HBc. The local ethics committee approved the study protocol and informed consents were obtained from all participating individuals.

### Cell cultures

Two human hepatocellular carcinoma cell lines HepG2 and HepG2.2.15 were grown in 1640 medium (GibcoBRL, USA) with 100 units/ml penicillin, 100 μg/ml streptomycin, and 10% heat-inactivated fetal bovine serum. Cells were grown at 37°C in a 5% CO_2 _incubator.

### Plasmid construction

HBV infectious clone pBlueks-HBV1.3 was constructed previously [15]. ApoM promoter was amplified from human genomic DNA using the following primers: 5' CATGGTACCATAAATTTATCAAGCTAGGTGT 3' (sense), 5' TACACGCGTCTTTCAGCTCCCTTGCGTTCG 3' (antisense), and then cloned into pGL3-Basic vector to generate ApoM promoter and Luciferase gene fusion plasmid(pAPOM-Luc). To create ApoM -encoding vector, the ApoM gene was amplified from HepG2 using the following primers: 5' TATGGATCCATGTTCCACCAAATTTGGGCA 3' (sense), 5' GCCAAGCTTTCAGTTATTGGACAGCTCACA 3' (antisense), and then cloned into pCMV-tag2B vector(Stratagene) to generate Flag2B-ApoM, in which ApoM protein was tagged by FLAG.

### Luciferase activity assay

HepG2 cells were seeded at density of 4.0 × 106 cells per 24-well plate or 6-well plate and grown to confluence reaching about 80%. pBluesk-HBV and pAPOM-Luc were co-transfected into the HepG2 cells using Lipofectamine 2000 (Invitrogen, Carlsbad, CA) according to the protocol provided by the manufacturer. Cells were serum-starved for 24 h before being harvested. Firefly and Renilla luciferase activities were measured as previously described[16]. Assays were performed in triplicate and expressed as means ± s.d. relative to vector control as a as 100(RLU) or luciferase activity (LUC).

### Semiquantitative RT-PCR analysis

Total RNA was isolated from HepG2 and HepG2.2.15 cells using Trizol reagent (Invitrogen, Carlsbad, CA) and reverse-transcribed with oligo (dT)_18 _primer. PCR reaction was performed in 25 μl solution with the gene-specific primers (20 μM) described as follows: ApoM sense: 5'-GCTCCCTGATGTTTCCCTGAC-3'; APOM anti-sense: 5'-CACGGCCCGAGATAAGACG-3'; β-actin served as an endogenous control using primers described previously[17].

### ELISA for ApoM measurement

**Figure 1:**
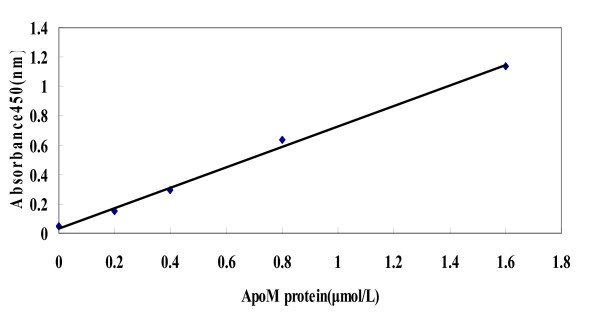
**Standard curve of ApoM protein was determined with two monoclonal antibodies, M42 and M58 by antigen capture ELISA**. Results were mean values from three separate plates run under same conditions.

ApoM levels in the culture media and serum were measured by sandwich ELISA based on two monoclonal antibodies, M42 and M58 which was used to quantify apoM, as described previously [18-20]. Each sample was tested in duplicate and the concentrations were determined from the standard curve (Figure 1).

### Assay for HBV protein expression

Forty-eight hours after infection, the level of HBs and HBe proteins in cell culture media was determined by enzyme-linked immunosorbent assay (ELISA) using HBV S antigen and HBV E antigen diagnostic kit (Shanghai KeHua Biotech Co. Ltd.), respectively.

### Analysis of HBV DNA by real-time PCR

To determine the effect of ApoM on HBV replication, intracellular core-associated DNA of HBV was extracted as described previously [21]. Briefly, cells were lysed and centrifuged at 25°C, and then magnesium chloride was added to the supernatant. DNA not protected by HBV core was digested with deoxyribonuclease (DNase I). After inactivating the DNase I, cell lysate was treated with proteinase-K and extracted with phenol/chloroform. Core-associated HBV DNA was recovered by ethanol precipitation and quantified by real-time PCR as described by the manufacturer (PG Biotech, Shenzhen, China). The HBV DNA in the supernatants was also quantified following the procedure provided by the manufacturer (PG Biotech, Shenzhen, China). PCR was carried out and analyzed by a PE Gene Amp 7700 (Perkin-Elmer).

### Statistical analysis

All statistical analyses were performed by SPSS 13.0 for windows (SPSS, Inc., Chicago). Results are presented as means ± SD. Group means were compared using the Student's t-test and Analysis of Variance (ANOVA). A P values of < 0.05 was regarded as statistically significant.

## Results

### Elevated apoM levels among chronic HBV infected patients

**Figure 2:**
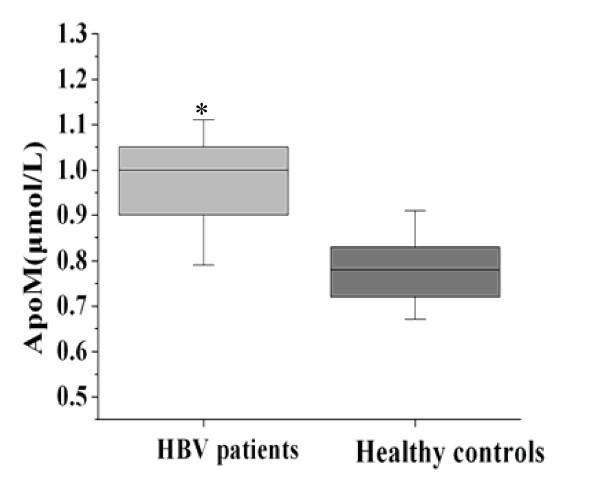
**Serum ApoM levels in patients and healthy individuals**. Serum ApoM levels were measured in 126 HBV-infected patients and 118 healthy individuals by ELISA. * *P *≤ 0.001.

We measured apoM concentrations in the sera of 126 HBV-infected patients and 118 healthy individuals. As shown in Figure 2, the average ApoM concentration was significantly elevated in these patients' sera compared to that of the control group (0.98 ± 0.10 μmol/L versus 0.77 ± 0.08 μmol/L; *P *≤ 0.001). ApoM levels were 27 percent higher in the patients' sera as compared to the healthy individuals (Figure 1).

### HBV enhances the ApoM promoter activity, the transcription of ApoM mRNA and its protein expression

**Figure 3:**
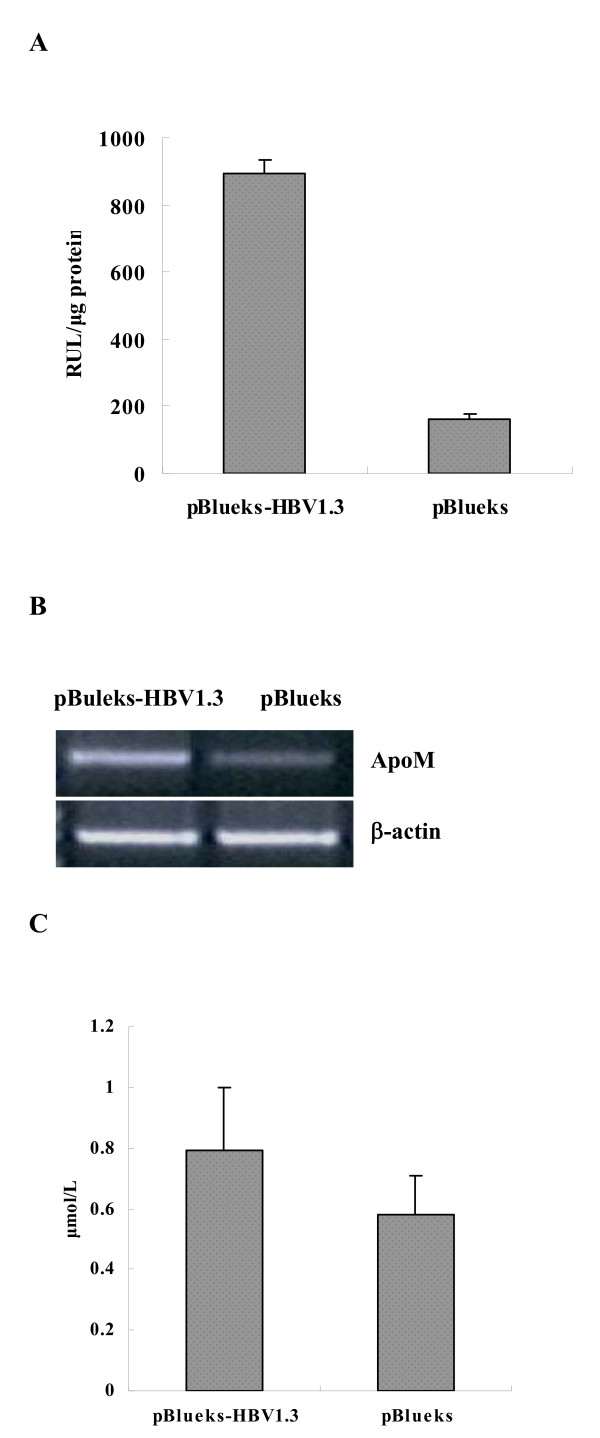
**Effects of HBV on ApoM expression in transfected cells**. (A) Effect of HBV on ApoM promoter activity. HepG2 cells were co-transfected with pGL3-ApoM-Luc and pBlueks-HBV1.3. PBlueks was used as vector control. Luciferase activities were determined and analyzed. (B) Effects of HBV on ApoM mRNA expression. HepG2 cells were transfected with pBlueks-HBV1.3 or pBlueks. ApoM mRNA was determined by semi-quantitative RT-PCR using primers specific to each of the genes, respectively. (D) Effects of HBV on ApoM protein expression. HepG2 cells were transfected with pBlueks-HBV1.3 or pBlueks. The levels of ApoM protein were determined by ELISA.

To determine the effects of HBV infection on ApoM expression, we measured the luciferase values by co-transfecting the reporter plasmid pAPOM-Luc and the infectious clone pBlueks-HBV1.3 or pBlueks (vector control) in order to evaluate the ApoM promoter activity in HepG2 cells. Results showed that ApoM promoter activity was raised by more than five folds (895 ± 37.8 RUL/μg *vs *162 ± 15.6 RUL/μg) (Figure 3A). These results demonstrated that HBV stimulated the activities of ApoM promoter. To examine the role of HBV on the activation of ApoM mRNA, we performed semi-quantitative RT-PCR. Total mRNA were extracted from HepG2 cells transfected with pBlueks-HBV1.3 or pBlueks. RT-PCR results showed that the levels of ApoM specific mRNA were significantly increased in the cells transfected with pBlueks-HBV1.3 as compared to pBlueks. The total mRNA was balanced by using beta-actin as an internal control (Figure 3B). These results suggest that HBV enhances ApoM mRNA expression. Comparable results were validated by ApoM protein expression in the presence or absence of HBV. ELISA results revealed that ApoM protein level was higher in HepG2 cells transfected with pBlueks-HBV1.3 than that with vector control (0.79 ± 0.21 μmol/L *vs *0.58 ± 0.13 μmol/L) (Figure 3C). These results demonstrated that HBV stimulates ApoM protein expression.

### Raised levels of ApoM suppress HBV protein expression and viral replication

**Figure 4:**
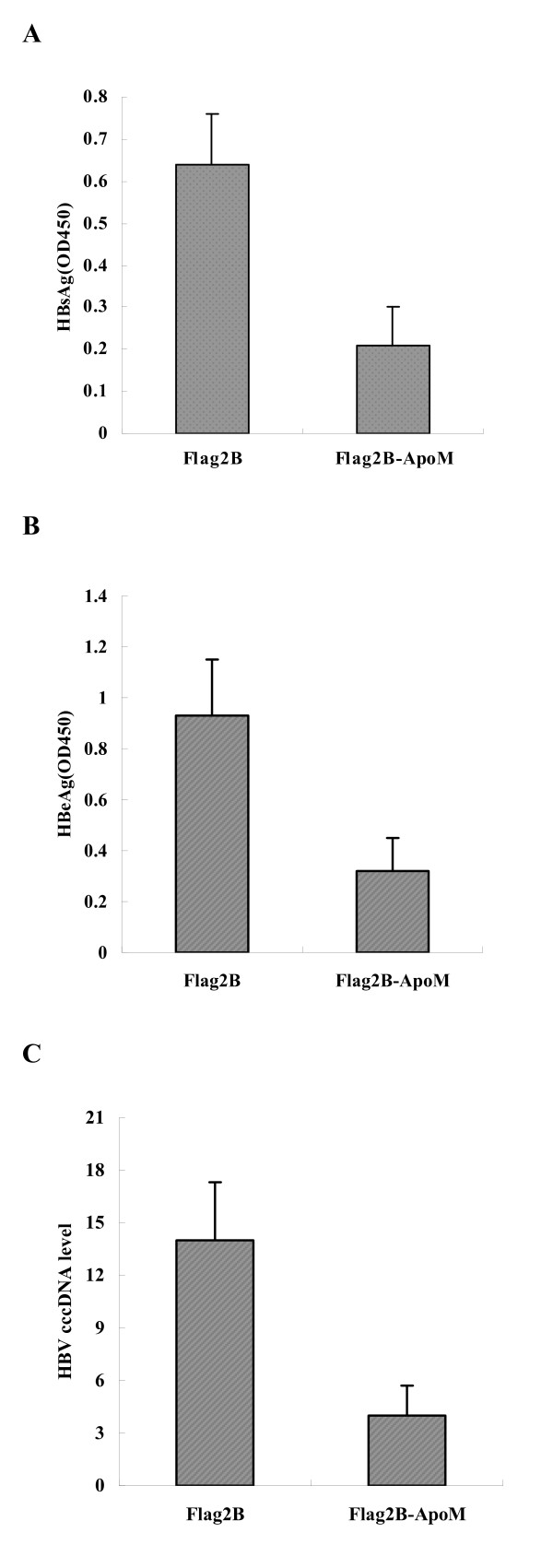
**Determination of the roles of ApoM in HBV gene expression and viral DNA replication**. HepG2.2.15 cells were infected with Flag2B-ApoM and harvested 48 h post-infection. The expression of S protein (A) and E protein (B) of HBV in the supernatant of cell culture was measured by ELISA using diagnostic kits for HBV. The production of core-associated DNA (C) of HBV was determined by real-time PCR. Results represent means of three independent experiments, with derived standard errors shown.

The increased levels of APOM in response to HBV should someway affect the virus. To evaluate the effects of ApoM on HBV gene expression and viral replication, HepG2.2.15 were transfected with Flag2B-ApoM or an empty vector control. Forty-eight hours post-infection, the levels S protein (HBs) and E protein (HBe) of HBV in the supernatant of cell cultures were measured by ELISA. Results showed that the level of HBs and HBe exhibited more than 3 folds decrease in ApoM over-expressing cells (Figure 4A and 4B), These results demonstrated that the expression of HBs and HBe proteins of HBV was suppressed by ApoM. The above results also implied that ApoM has a inhibitory effect on HBV replication. To confirm such effect, the levels of intracellular core-associated DNA of HBV were determined by real-time PCR. Results showed that the level of HBV DNA was decreased more than 3 folds in ApoM over-expressing cells (Figure 4C). These results clearly indicated that ApoM can inhibit HBV replication.

## Discussion

HBV is not directly cytopathic but its interaction with the host immune system creates opportunity for HBV DNA integration into the host genome. HBV causes liver injury by an immune response against the virus-infected liver cells[22,23].

ApoM was originally found in chylomicrons and is found in all major lipoprotein classes [9], it is highly hydrophobic and must co-circulate with lipoprotein particles in the blood stream[24,25]. In this study we present evidence that HBV promotes ApoM production both in vivo and in vitro.

A 27 percent increase in ApoM levels in HBV patients as compared to healthy individuals was observed. It is well known that chronic liver diseases can interfere with hepatic metabolism of lipids, lipoproteins or apolipoproteins, as most plasma endogenous lipids and lipoproteins are synthesized by the liver, which depends on the integrity of liver cell function. As apoM is exclusively expressed in hepatocytes and kidney tubular cells [26,27], it could be hypothesized that the normal hepatic physiological processes are necessary for hepatic apoM production, and in cases of HBV infection, there may be interference in the apoM synthesis. It has been reported that the plasma apoM levels were higher in the patients suffered from HCC and other chronic liver diseases than in normal subjects[28,29]. Our result is in line with earlier results from experiment based on semiquantitatively determined by both dot-blotting and western blotting analysis[29]. However, we didn't find any correlation between HBV viral load and ApoM levels.

Initial rise in HBV serum ApoM prompted us to investigate further. Our in vitro results showed that there was a six fold increase in the promoter activity of the ApoM in the presence of infections HBV clone. This increase in ApoM promoter activity was substantiated by increased ApoM mRNA level and protein expression. Many intracellular pathways are activated by HBV infection, which may positively regulate ApoM. The cellular signaling pathways involved in the process are currently under investigation.

A rise in the ApoM protein expression in response to viral infection poses a potential query that how this would affect the contributory viral agent. To respond to this issue, we further studied the effects of ApoM on HBV gene expression and viral replication in cell culture models by using HepG2.2.1.5 cell line, which carries several copies of the HBV genome on its chromosome. The effects of ApoM on HBV gene expression and viral replication were studied thoroughly by the analyzing the levels of viral protein production through enzyme-linked immunosorbent assays and the levels of viral RNA expression by semi-quantitated RT-PCR analysis. All results indicated that ApoM had significant inhibitory effects on viral mRNA expression, viral protein production. Thus, we provided evidence that ApoM is an important mediator of HBV replication and may be a potential therapeutic agent for HBV infection.

In a nutshell, our present work shows that raised ApoM levels in HBV infection may in turn hamper HBV replication in vitro, one of the protective mechanisms of nature during HBV infection. However, whether apoM in vivo can protect against the HBV infection is still unknown, experiments showing the dose response effect of HBV infection in apoM genemodified mice were to be done.

## Competing interests

The authors declare that they have no competing interests.

## Authors' contributions

CLZ participated in the assays of luciferase activity and HBV protein expression. DZC performed the statistical analysis. YX participated in the plasmid construction and immunoassays. FL and XZ participated in its design. All authors read and approved the final manuscript.
